# Giant Brunner's gland hamartoma causing retrograde jejuno-duodenal intussusception: A case report

**DOI:** 10.1016/j.amsu.2018.12.007

**Published:** 2019-01-04

**Authors:** Mohammed Yousef Aldossary, Ali A. Alzahir, Liqa A. Almulla, Zahrah H. Alhajji, Osama H. Alsaif

**Affiliations:** a Department of General Surgery, Surgical Oncology Section, King Fahad Specialist Hospital-Dammam, Saudi Arabia; b Department of Pathology and Laboratory Medicine, Histopathology Consultant, King Fahad Specialist Hospital-Dammam, Saudi Arabia

**Keywords:** Brunner's gland, Hamartoma, Retrograde, Intussusception, Duodenum

## Abstract

Brunner's gland hamartoma is a rare benign duodenal tumor. It occurs in Brunner's glands, which are found in the duodenum and produce secretions that protect the duodenum from pancreatic enzymes, gastric acid, and other agents. Endoscopic or surgical resection is required for these hamartomas. Duodenal intussusception is a relatively rare condition, usually caused by the presence of benign tumors, such as fibroadenomas, lipomas, papillomas, or sometimes with malignant neoplasms.

We report a case of giant Brunner's gland hamartoma in the duodenum causing antiperistaltic intussusception in a 45-year-old female patient. The patient reported a 3-year history of chronic anemia, and this mass was detected incidentally by computed tomography (CT) during investigations for chronic anemia and weight loss. Pre-operative abdominal and pelvis contrast revealed a sausage-shaped intraluminal structure with alternating fat planes and vessels distended in the third part of the duodenum up to the first part of the duodenum. Pancreas-sparing duodenectomy was performed. The patient recovered very slowly and was discharged on postoperative day 15 in good condition. Histology showed a large polypoid mass measuring 12.0 × 7.5 × 2.0 cm^3^, consistent with Brunner's gland hamartoma.

Brunner's gland hamartoma can present with features of duodenal intussusception or ampullary obstruction but is rarely seen to cause retrograde jejuno-duodenal intussusception. Pancreas-sparing duodenectomy is the best surgical option in adult patients with intestinal intussusception associated with giant lesions close to the ampulla of Vater, especially in the presence of features of malignancy.

## Highlights

• Brunner's gland hamartoma is a rare benign duodenal tumor.
• Adult intussusception is usually caused by benign or malignant tumors, but rarely to be caused by Brunner's gland hamartoma.
• Pancreas-sparing duodenectomy is the surgical option for lesions close to the ampulla of Vater.

## 1 Introduction

This work has been reported in line with the SCARE criteria [1].

Brunner's gland hamartoma is a rare benign tumor of the duodenum, which is also known as Brunneroma or Brunner's gland adenoma [2]. This tumor occurs in the Brunner's glands, which are found in the duodenum and produce secretions that protect the duodenum from pancreatic enzymes, gastric acid, and other agents [3]. These tumors are usually asymptomatic and only diagnosed incidentally.

Intussusception occurs when one segment of the bowel slides into the lumen of an adjacent bowel segment [4,5]. Adult intussusception is a rare condition representing 5% of all cases of intussusception [[6], [7], [8]]. Adult intussusception is classified into two types: anterograde and retrograde [4]. Anterograde intussusception is the most common, whereas retrograde intussusceptions are very rare and represent 1% of all cases [4]. There are a few cases reported in the literature regarding jejunoduodenal intussusception; the primary etiology was post-operative complications from gastric bypass or placement of gastrostomy tubes, resection of jejunal duplication cyst, or duodenojejunostomy [[9], [10], [11], [12], [13]]. According to our knowledge, there are only few cases in the literature of duodenojejunostomy for retrograde intussusception caused by a giant Brunner's gland hamartoma. Our case is the second largest Brunner's gland hamartoma reported.

## 2 Case report

A 45-year-old woman with a 3-year history of chronic anemia was followed up at the Gastroenterology outpatient department of another hospital, with a 6-month history of abdominal pain. The pain was intermittent, worse in the mornings, and localized to the epigastric region. The patient noticed that her abdomen became distended after meals and distension was relieved after a bowel movement. The patient noticed a 12-kg weight loss over the last 6 months. There was a history of nausea but no vomiting. The patient denied any history of loss of appetite, diarrhea or constipation, and upper or lower gastrointestinal bleeding. There was no significant past medical history other than anemia and multiple blood transfusions. The patient denied any history of tobacco smoking, alcohol consumption, drug abuse, *Helicobacter pylori* (*H. pylori*) infection, chronic renal failure, peptic ulcer disease, and chronic pancreatitis. Her family history was unremarkable. The patient did not have any prior surgeries or history of malignancy. Workup for her chronic anemia included CT scan of the abdomen and pelvis with intravenous and oral contrast, which revealed duodenal intussusception. The patient was booked for upper gastrointestinal endoscopy in the referral hospital but missed her appointment and asked for a referral to another hospital.

**Fig. 1:**
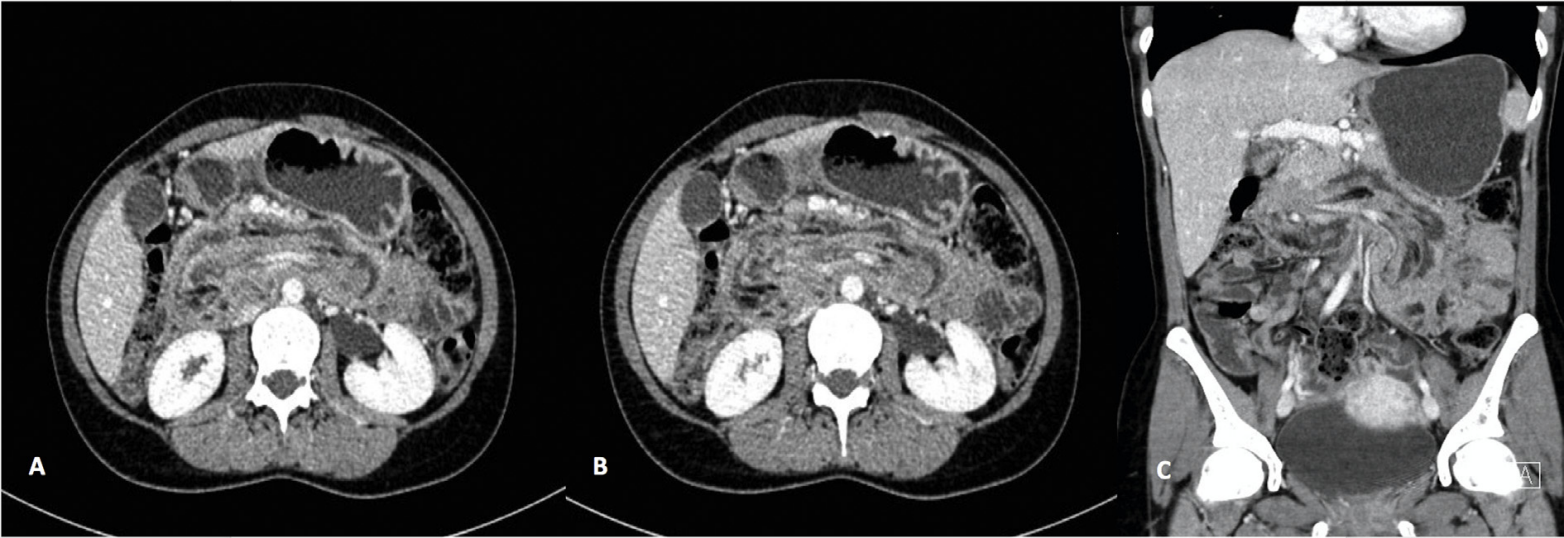
A and B: CT scan of abdomen and pelvis with contrast axial views reveals a significantly dilated stomach with no detectable gastric lesion. The third part of the duodenum is distended by a sausage shaped intraluminal structure with alternating fat planes and dilated vessels reaching up to the first part of the duodenum in keeping with retrograde jejuno-duodenal intussusception. There are no detectable soft tissue lesions and the rest of the bowels are unremarkable. C: Coronal view with the same findings.

Upon arrival at our hospital, she appeared underweight (height, 162 cm; weight, 43 kg). Her vitals were stable, and she was afebrile (blood pressure, 118/67 mm/Hg; heart rate, 96 bpm; respiratory rate, 23 bpm; oxygen saturation, 98% in room air; temperature: 36.9 °C). On examination, her abdomen was soft and laxative, with no tenderness, or organomegaly. A complete blood count on the day of admission revealed the following: low hemoglobin level, 7.2 g/dL (12.0–17.0 g/dL); mean corpuscular volume (MCV), 78.0 fL (80.0–100.0 fL); low hematocrit, 24.8% (40.0%–51.0%); leukocyte count, 8.2 × 10^9^/L (4.5-11.0 × 10^9^/L); and platelet count, 323 × 10^9^/L (150-450 × 10^9^/L). Other laboratory profiles were unremarkable. Contrast CT of the abdomen and pelvis revealed significant dilation of the stomach with no detectable gastric lesion. The third part of the duodenum was distended by a sausage-shaped intraluminal structure with alternating fat planes and dilated vessels reaching up to the first part of the duodenum, suggesting retrograde jejuno-duodenal intussusception. There was no detectable soft tissue lesion, and the rest of the bowel was unremarkable (Fig. 1A–C). Endoscopic examination of the upper digestive tract revealed a large, long, mobile submucosal longitudinal and tortuous bulge extending from the first to the second part of the duodenum that moved freely with bowel contraction. The papilla of Vater was normal with no neoplastic process.

**Fig. 2:**
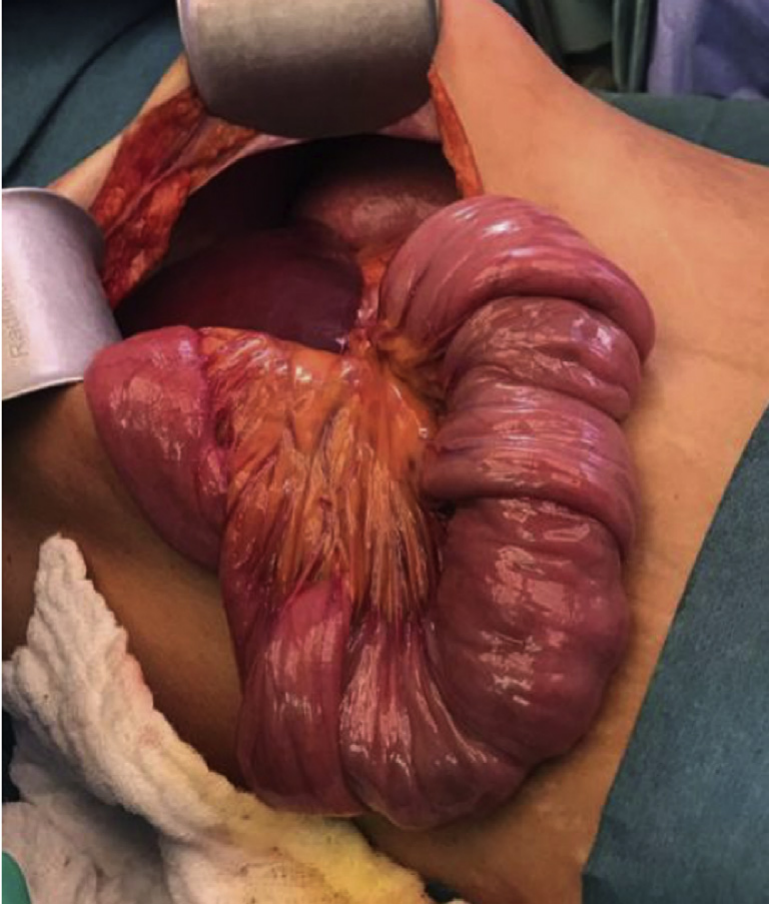
Retrograde intussusception in the proximal jejunum.

**Fig. 3:**
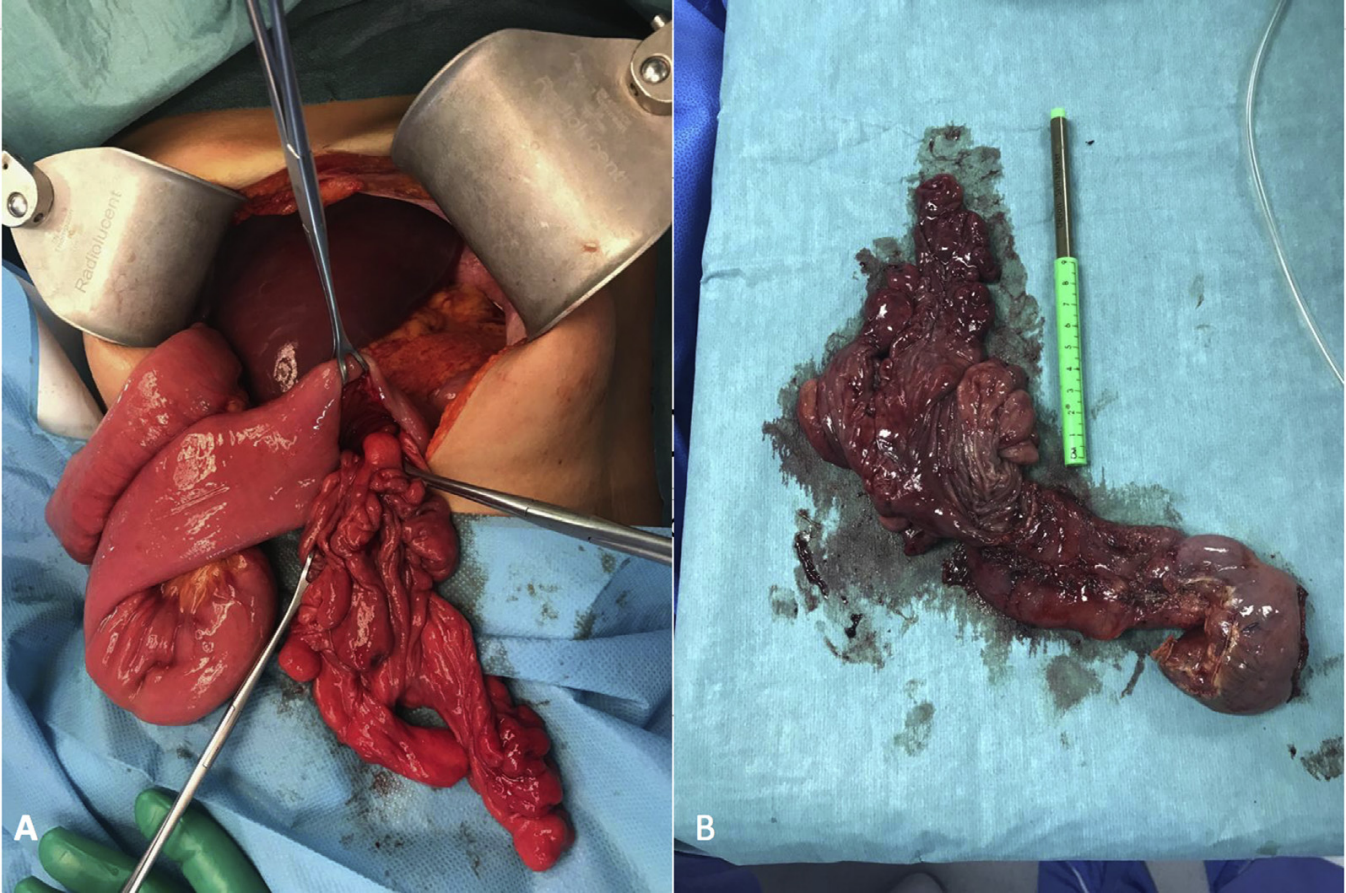
A: Jejunostomy with mass pulled through it. B: Duodenum with the mass after resection.

The patient underwent laparoscopic exploration, which revealed antiperistaltic intussusception in the proximal jejunum (Fig. 2). A jejunostomy was performed, and the mass was pulled through in the proximal jejunum (Fig. 3A).

A cholecystectomy was performed, and a pediatric feeding tube was passed through the cystic duct to locate the ampulla of Vater. The mass was not resectable without affecting the ampulla, so the decision was made to perform a pancreas-sparing duodenal resection. The excised duodenum with mass was sent for histopathological examination (Fig. 3B).

**Fig. 4:**
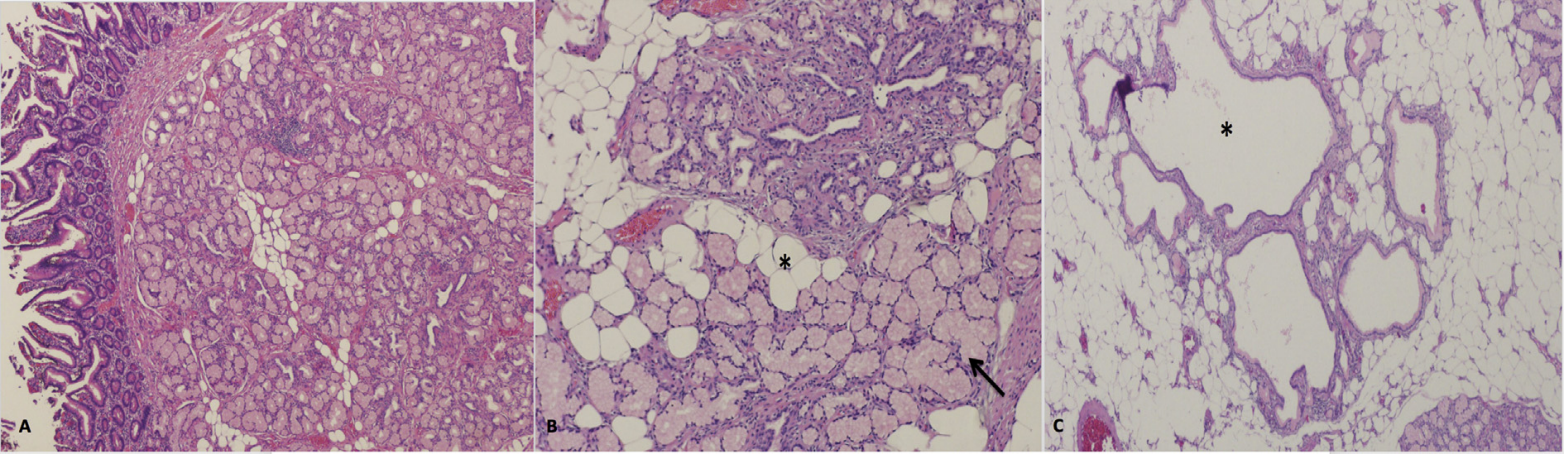
A: Low power view shows submucosal Brunner glands proliferation separated by fibrous stromal bands. B: On high power, the benign Brunner's glands (arrow) are intermingled with adipocytes (asterisk). C: The proliferating Brunner's glands are accompanied by cystically-dilated ducts (asterisk) and adipose tissue with no microscopic features of malignancy.

Pathological findings showed a large polypoid mass with the mucosa pedunculated from the bowel lumen, measuring 12 × 7.5 × 2.0 cm^3^. Microscopic examination revealed a bowel mucosa with underlying submucosa showing lobules of benign normal-appearing Brunner's glands separated by fibrous stroma. The nodular proliferation of Brunner glands was accompanied by cystically dilated ducts and adipose tissue with no microscopic features of malignancy (Fig. 4A–C). The patient was discharged postoperatively in good condition, given an appointment after 1 month.

## 3 Discussion

Brunner's gland hamartoma is an uncommon benign tumor of the duodenum representing 5–10% of all benign duodenal tumors, with an overall incidence rate <0.01% [14]. Most tumors are asymptomatic, small, and identified incidentally [2]. Sometimes the lesions lead to duodenal or ampullary obstruction, intussusception, and gastrointestinal bleeding [[15], [16], [17]].

Retrograde intussusception is an invagination or sliding of a distal segment of the bowel (the intussusceptum) into the receiving proximal end of the bowel (the intussuscipiens) [18]. The pathogenesis of retrograde intussusception is unknown [18]. However, it is suggested to be caused by reverse peristalsis secondary to distal obstruction in the presence of a fixed bowel, which is typically caused by an extrinsic neoplastic or inflammatory processes [4].

In our case, retrograde intussusception was caused by Brunner's gland hamartoma, which triggered antiperistaltic activity.

Since its first description in the 19th century, <200 cases of Brunner's gland hamartoma have been reported in the medical literature [19]. Interestingly, Brunner's gland hamartoma usually occurs in middle-aged persons and is equally incident in men and women [20]. The size of most lesions is 1–3 cm; however, several cases reported lesions >5 cm. The largest reported lesion was 12 cm [21]. Nearly 57% of these tumors are located in the duodenal bulb, 27% are found in the second part of the duodenum, and 7% in the third part of the duodenum. Less frequently, these tumors may occur in the pyloric canal (5% of cases), proximal ileum (2%), and jejunum (2%) [22].

The exact pathogenesis of Brunner's gland hamartoma is unknown. A theory is that hyperacidity causes hyperplasia of Brunner's glands; however, only 45% of patients with this condition had increased gastric acid secretion and 20% of patients with Brunner's gland hyperplasia had gastric hypoacidity, making this theory unlikely [21]. There is also a theory that concurrent *H. pylori* infection, which is very common in patients with Brunner's gland hamartoma, causes this condition [23]. However, there is no understanding of its role in the pathogenesis of Brunner's gland hamartoma, and our patient had no history of H. Pylori infection.

Medical professionals have also suggested that this condition may be associated with chronic renal failure, peptic ulcer disease, and chronic pancreatitis [24]. Our patient denied any history of those diseases.

The diagnostic modalities of Brunner's gland hamartoma are imaging and endoscopy. Nevertheless, to arrive at a definitive diagnosis it is necessary to conduct histopathological examination [1].

According to Hirasaki et al. [20], the best way to diagnose Brunner's gland hamartoma is by combining endoscopic and radiographic findings: the radiographic finding indicative of a duodenal tumor is a sessile or pedunculated polypoid filling defect, while the corresponding endoscopic findings may include a pedunculated polyp or sessile, lobulated or polypoid mass covered with normal mucosa.

When formulating a diagnosis, it is important to distinguish Brunner's gland hamartoma from other types of duodenal lesions such as polypoid adenoma of the superficial mucosal glands, leiomyoma, malignant tumors, and aberrant pancreatic tissue [20]. Endoscopic biopsies are rarely sufficient for a conclusive diagnosis because in many cases these biopsies are not deep enough to reach the submucosal tumor tissue [17]. The final diagnosis can be reliably established after analyzing the pathological findings of resected specimens that are derived from surgical treatment, polypectomy, or endoscopic mucosal resection [20].

One of the most common treatments of Brunner's gland hamartoma is endoscopic removal. However, if unsuccessful, surgical excision is required [24]. In most cases, endoscopic removal works well because Brunner's gland hamartoma is considered histologically and clinically benign. However, it is important to keep in mind that several cases of malignancy arising from Brunner's glands were reported [25]. In children, intussusceptions are usually primary or idiopathic, whereas most cases of adult intussusceptions are caused by a structural lesion [26]. A significant percentage of these masses are malignant neoplasms, which account for up to 66% of colonic intussusceptions and 30% of small intestine intussusceptions [26]. In our case, the mass was attached to the ampulla of Vater and we performed a pancreas sparing duodenectomy, being careful to obtain negative margins. The potential for malignancy was considered because of the history of chronic anemia, significant history of weight loss, and as the most common cause of intestinal intussusception in adults is a malignancy. Pancreas-sparing duodenectomy has been widely used for various duodenal benign and malignant tumors [27,28]. Advantages of this procedure are restoration of gastrointestinal tract continuity and preservation of organs such as the head of the pancreas, leading to a better long-term outcome. Mackey et al. [29] found that pancreas-sparing duodenectomy is a safe procedure for treating patients with duodenal polyposis. In addition, Sarmiento et al. [30] found that pancreas-sparing duodenectomy in patients with duodenal polyposis results in good quality of life, excellent absorptive capacity, and weight gain.

## 4 Conclusion

Brunner's gland hamartoma can present with the features of duodenal intussusception or ampullary obstruction, but it is rarely seen to cause retrograde jejuno-duodenal intussusception. For giant lesions close or attached to the ampulla of Vater, a pancreas-sparing duodenectomy is the best surgical option, especially if the patient has features of malignancy. There is need for further research into the pathogenesis of Brunner's gland hamartoma, as our case did not support any of the current hypotheses on the pathogenesis.

## Ethical approval

Ethical approval was obtained from the Institutional Review Board (IRB) of the King Fahad Specialist Hospital, Dammam, Saudi Arabia. The ethical approval was signed on 06th November 2018.

## Sources of funding

This study did not receive any funding from governmental or private organizations.

## Author contribution

All authors MYD, AAA, LAA, ZHA, OHA participated in Concept, data collection, and Literature search. Writing of the paper were made by MYD and OHA. Histopathological part and images were performed by LAA. All authors read and approved the final manuscript.

## Conflicts of interest

The authors declare no conflict of interest.

## Trial registry number

N/A.

## Guarantor

Dr. Mohammed Yousef Aldossary.

## Consent

Written informed consent was obtained from the patient for publication of this case report and accompanying images.

## Provenance and peer review

Not commission, externally peer reviewed.
